# Label-free detection of microRNA based on coupling multiple isothermal amplification techniques

**DOI:** 10.1038/srep35982

**Published:** 2016-10-25

**Authors:** Xiangjiang Zheng, Li Niu, Di Wei, Xuemei Li, Shusheng Zhang

**Affiliations:** 1 Key Laboratory of Sensor Analysis of Tumor Marker, Ministry of Education, College of Chemistry and Molecular Engineering, Qingdao University of Science and Technology, Qingdao 266042, P. R. China; 2 Shandong Provincial Key Laboratory of Detection Technology for Tumor Makers, Research Institute of Biochemical Analysis, Linyi University, Linyi 276000, P. R. China

## Abstract

MicroRNA (miRNA) was a promising class of cancer biomarkers. Here we developed a label-free method for sensitive measurement of let-7d miRNA based on multiple amplification techniques. The primer will bind to the duplex strand DNA that was formed by stem-loop template and target let-7d to initiate strand displacement amplification (SDA) in tandem. The released single strand DNA will be a primer to bind the circular template to initiate rolling circle amplification (RCA). The products based on multiple amplifications will be detected by a standard fluorimeter with N-methyl mesoporphyrin IX (NMM) as the fluorescent indicator. The proposed method exhibited excellent selectivity and high sensitivity with a detection limit of as low as 1.5 × 10^−13^ M. Moreover, this methodology was used for the determination of biomolecules in real serum samples with satisfying results.

MicroRNA was a family of small and non-coding RNAs that was 18–25 nucleotides in length^1^. More and more evidences indicated that miRNA participated in the gene expression and aberrant expression that was related to cancer initiation and tumor stage in human tissues and blood samples^2^,^3^,^4^, highlighting their potential as both diagnostic biomarkers and drug targets^5^,^6^,^7^.

Detection of miRNA has attracted great interests. Up to now, many methods have been developed. Northern blotting was the standardized method to detect miRNA^8^, but it suffers from time-consuming operations. Microarray was an effective method for high-throughput detection of miRNAs^9^, however it needs miRNA labeling and probe fixation. The quantitative real-time polymerase chain reaction (qRT-PCR) was another conventional method with high sensitivity^10^, however it faces challenges of efficient binding of shorter primer and miRNA templates. Surface-enhanced Raman scattering methods were developed^11^,^12^, but it is difficult to accurately quantify. Biosensing methods based on nanomaterials are still limited to complex operation, expensive label, and laborious synthesis^13^,^14^,^15^.

The low abundance of miRNA in human biological samples make them difficult to analyze based on traditional methods^16^. Recently, signal amplifying methods based on enzyme reactions have been developed, such as loop-mediated isothermal amplification^17^, exponential amplification reaction^18^,^19^, rolling circle amplification (RCA)^20^,^21^, strand displacement amplification (SDA)^22^,^23^. SDA and RCA are isothermal DNA amplification techniques based on enzymes. They were power and essential tools for realizing detection of target by translating diversified targets (such as DNA, RNA or protein) into input DNA before subsequent DNA replication^24^. However it is difficult to get a good sensitivity to only use simple amplification. Zhang *et al*.^25^ have developed a bifunctional SDA-mediated hyper-branched rolling circle amplification method to detect miRNA, but it needs higher temperature (62 °C) to realize the reaction.

We have developed a label-free method to detect miRNA based on strand displacement amplification and rolling circular amplification. To investigate whether SDA-mediated RCA reaction could effectively improve the specificity of miRNA detection, let-7 family members with high sequence homology were chosen to be detected by this method. Reduced expression of let-7 miRNA in different human cancer tissues or cells is accompanied by the fluctuation of correlative target genes expression, indicating that let-7 miRNA is closely linked to cancer^26^,^27^.

The principle of measurement of let-7d is illustrated in Fig. 1. The template **S**_**1**_contains a stem-loop structure. Target let-7d could open the stem-loop structure and form duplex structure. The primer **S**_**2**_will hybridize with the DNA duplex and initiate a chain polymerization reaction with the help of Klenow Fragment (exo-) polymerase. When there is no target, the stem-loop structure will not be opened by the primer and the chain polymerization reaction will not take place. There is a cleavage cite of nicking endonuclease Nb.BbvCI in the polymerized duplex chain. The duplex chain will be cleaved with the help of Nb.BbvCI. A new polymerization will take place and the cleaved single strand DNA (**S**_**4**_) will be released. **S**_**4**_ will hybridize with the template **S**_**5**_ (which owns the cleavage cite of Nb.BbvCI) and initiate a new polymerizing/nicking amplification, producing single strand DNA **S**_**6**_. The released single strand DNA **S**_**6**_ will be a new primer to hybridize with circular template (CT) and initiate rolling circle replication and produce single long strand DNA with the help of phi29 DNA polymerase. There are many guanine bases in the single long strand DNA strands and G-quadruplex structure will be formed with potassium ions as stabilizer^28^,^29^.

N-methyl mesoporphyrin IX (NMM), N-core methylated non-planar derivative of mesoporphyrin IX, is a natural porphyrin and first identified as a potent inhibitor of ferrochelatase which is involved in heme synthesis^30^. The chemical structure diagram of NMM is in Fig. S4. NMM is an anionic porphyrin with a good structural selectivity for G-quadraplex^31^,^32^,^33^. It has weak fluorescence signal by itself, but exhibits a dramatic fluorescence enhancement after binding to G-quadruplex. There is no similar phenomenon upon binding to duplexes, triplexes, or single-stranded forms. We used NMM as fluorescence indicator to detect fluorescence of products.

## Results and Discussion

### Feasibility Analysis of the method

To better investigate the SDA reaction, single strand DNA **SF** that has a stem-loop structure was synthesized. **SF** was decorated by fluorophore-FAM and quenching group-BHQ1 at the two ends each other. **SF** has no fluorescence signal because of the quenching function of BHQ1 to FAM. The single strand DNA **S**_**6**_ will bind to **SF** and open the stem-loop structure and realize the fluorescence signal on. The concentration of ssDNA **S**_**6**_can be detected based on fluorescence intensity of SF (See Figs S1 and S2).

**Figure 2:**
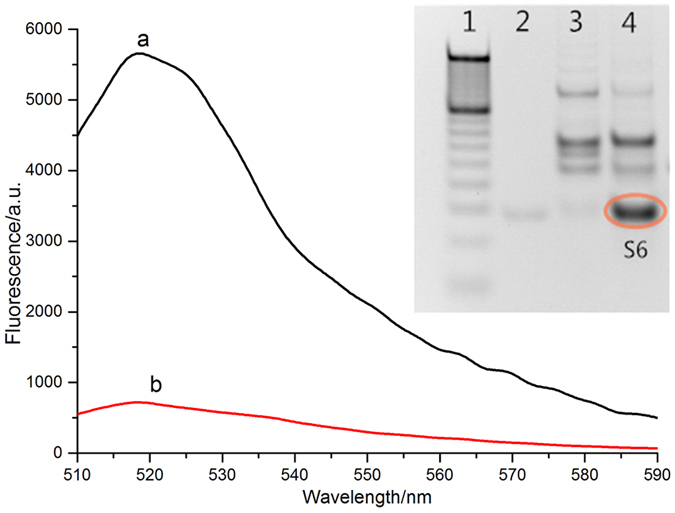
Fluorescence intensity of amplification products by SDA in the presence (**a**) and absence (**b**) of let-7d. The concentration of target let-7d (curve a) is 10 nM. Inset: native polyacrylamide gel electrophoresis (10%) of amplification products of SDA: lane1 100 bp marker, lane 2 only S_6_ that was synthesized as a standard comparation, lane 3 absence of target, lane 4 presence of target.

The SDA reaction was confirmed by measurement of fluorescent spectra and gel electrophoresis. In Fig. 2, the fluorescence intensity of curve b was almost unchanged, indicating that no reaction occurred. But in curve a, the fluorescence intensity gradually increased, indicating that let-7d initiated the SDA reaction and produced a large number of DNA products (**S**_**6**_). It was also confirmed in native polyacrylamide gel electrophoresis. Lane 4 in the inset of Fig. 2 showed the amplification products **S**_**6**_ by SDA reaction in the presence of let-7d. In the same horizontal location, there was almost no **S**_**6**_ signal at lane 3, indicating no reaction occurred in the absence of let-7d.

To better investigate the products of multiple amplification, agarose gel electrophoresis was also conducted. In the Fig. S3, the products based on different concentration of target were demonstrated and the products would not nearly move in the agarose gel electrophoresis with 10 K ladder as a marker. The reason is that the products of RCA will be longer than 10000 bases. In addition, different concentration of products can be discriminated based on the gray level of products.

### The Optimization of Experimental Condition

**Figure 3:**
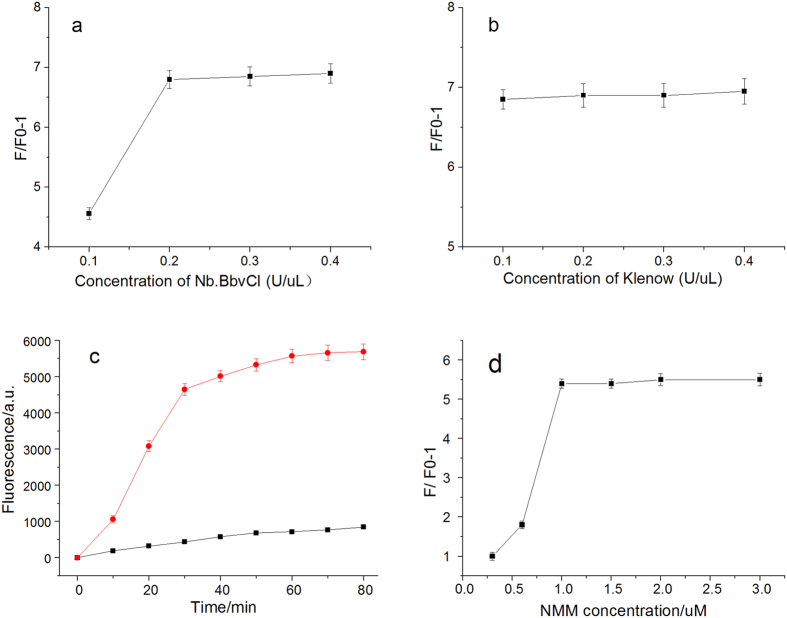
(**a**) Fluorescence response of different concentration of Nb.BbvCI in SDA reaction (**b**) Fluorescence response of different concentration of Nb.BbvCI in SDA reaction. (**c**) The time-dependent fluorescence response in SDA reaction. (**d**) Fluorescence intensity of different NMM concentration in RCA reaction.

The concentration of nicking endonuclease Nb.BbvCI is an important factor that affects the amplification reaction. The fluorescence signals were analyzed and data were showed in Fig. 3a. The variance of the (F–F0)/F0 value with the concentration of Nb.BbvCI, where F and F0 are the fluorescence intensity at 518 nm in the presence and in the absence of target miRNAs, respectively. The fluorescence signals increased with increasing concentration of Nb.BbvCI. But there is nearly no change of fluorescence intensity when the concentration increased from 0.2 U/μL to 0.4 U/μL.

The effect of the amount of Klenow Fragment exo- DNA polymerase on the assay was assessed (see Fig. 3b). Though the amount of polymerase was changed from 0.1 U/μL to 0.4 U/μL, there was little change in the fluorescence signals. Different reaction time of SDA was investigated. The data were shown in Fig. 3c. In order to obtain optimal detection sensitivity, 0.1 U/μL Klenow Fragment (exo-) polymerase, and 0.2 U/μL Nb.BbvCI nicking enzyme, the reaction time of 60 min were selected, respectively.

The concentration of fluorescence indicator NMM in RCA reaction was investigated. The data was shown in Fig. 3d. The concentration of 1.0 μM was selected as the optimal concentration of NMM.

### Sensitivity of miRNA Assay

**Figure 4:**
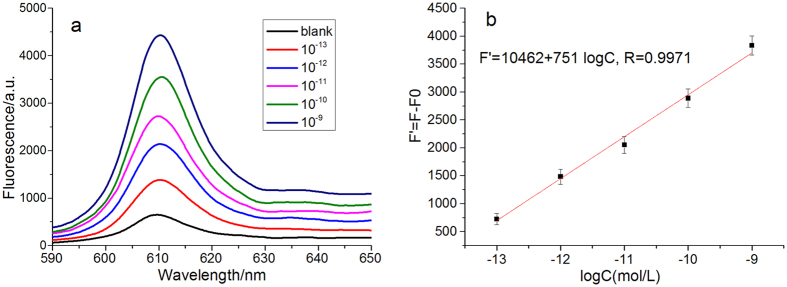
(**a**) Fluorescence spectra of NMM based on multiple amplification techniques under different concentration of target miRNA. (**b**) Standard working curve of different concentration miRNA.

Under the optimal condition, different concentrations of let-7d were detected. As shown in Fig. 4, the fluorescence increases with the increase of let-7d concentration. The fluorescence intensity has a linear correlation with the logarithm of let-7d concentration from 10^−13^ to 10^−9^ M. The standard curve equation is F’ = 10462 + 751 logC with a correlation coefficient of 0.9971, where C and F’ are the concentration of let-7d and the fluorescence intensity (F’ = F-F0), respectively. The detection limit is calculated to be 1.5 × 10^−13^ M, according to the 3σ rule, which is much lower than that of the existing signal amplification in our previous report^34^. Moreover, the result was comparable with other amplification methods and the comparison of different methods for miRNA detection is shown in Table S1. These results demonstrate the amplification efficiency of cascade recycling strategy and reveal that the proposed biosensor is efficient for ultrasensitive detection of miRNA.

### Specificity of miRNA assay

There was significant sequence homology among miRNA family members, especially let-7 miRNA family. It is difficult to discriminate them because there are highly homologous and only one or several difference bases.

**Figure 5:**
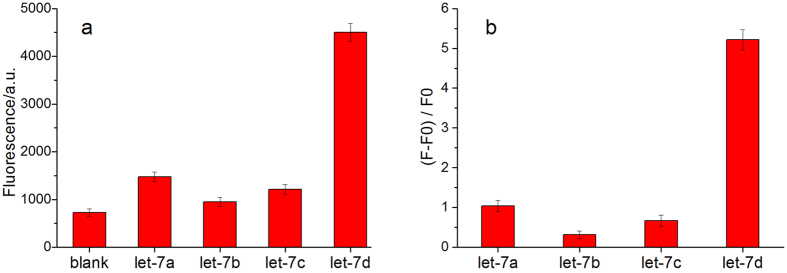
Specificity of miRNA assay. (**a**) Fluorescence intensity of let-7 family on the same concentration (**b**) Calculation of (F-F0)/F0 according to chart (**a**).

There is only two bases mismatch between let-7d and let-7a, three bases mismatches between let-7d and let-7b, four bases between let-7d and let-7c. The detection specificity of these let-7 family members were investigated under the optimal condition. As shown in Fig. 5b, according to the calculation of (F-F0)/F0, the fluorescence intensity of let-7d is 5.0-fold higher than that of let-7a, and 16.8-fold higher than that of let-7b, and 7.8-fold higher than that of let-7c, suggesting the high specificity of the proposed method for miRNA assay.

### Application in real sample

To investigate the applicability of this method in real samples, experiments were carried out in human serum samples. Various concentrations of let-7d were added in dilution solution of human serum samples that were obtained from Linyi Cancer Hospital and diluted 500-fold with DEPC treated water. The data shown in Table 1 exhibited good recovery and indicated great potential for specific detection of let-7d in serum samples.

## Conclusions

Taken together, we have developed a label-free method for accurate sensing of low-abundance miRNA molecules based on SDA-mediated RCA amplification techniques in tandem, which enables facile and reliable differentiation between let-7d and other let-7 family. High sensitivity was achieved through enhanced fluorescence signal of G-quadruplex with NMM as fluorescence indicator, with a detection limit of 1.5 × 10^−13^ M. The proposed method with high sensitivity and excellent selectivity, provided significant prospect for practical application.

## Materials and Methods

### Reagents and chemicals

All oligonucleotides except for circular template were HPLC purified and obtained from Sango. Biotech (Shanghai, China). Circular template was obtained from TaKaRa Bio. Inc (Dalian, China). The Klenow Fragment (exo-) DNA polymerase and phi29 DNA polymerase were purchased from Thermo Fisher Scientific Inc. The nicking endonuclease of Nb.BbvCI and the corresponding buffers (such as NE buffers 2) were purchased from New England Biolabs Inc.

The DEPC-water (Rnase free water) was purchased from Generay Biotech. Inc. NMM was obtained from Frontier Scientific Inc. The deoxynucleotide mixture (dNTPs) and 4S Red Plus Nucleic Acid Stain and Rnase Inhibitor were obtained from Sangon Biotech.

Nucleic Acid Sequence (5′-3′):

S_1_: CAA TTC CTC AGC GGA TGA GGT AGG CTA TGC AAC CTA CTA CCT CAT CCG CTG-NH_2_

S_2_: CAG CGG AT

S_5_: GTC AGA TGA ATT CGT GTG AGC CTC AGC CAA TTC CTC A

S_6_: TGA GGC TCA CAC GAA TTC ATC TGA C

SF: FAM-CAG AAC CTG TGT CAG ATG ACT TCG TGT GAG CCT CTA CAC AGG TTC TG-BHQ1

Circular Template (CT): P-ATT CGT GTG AGA AAA CCC AAC CCG CCC TAC CCA AAA GTC AGA TGA

Let-7a: **U**GA GGU AGU AGG UUG **U**AU AGU U

Let-7b: **U**GA GGU AGU AGG UUG **UG**U **G**GU U

Let-7c: **U**GA GGU AGU AGG UUG **U**AU **G**GU U

Let-7d: AGA GGU AGU AGG UUG CAU AGU U

### SDA-RCA amplification Reaction

All oligonucleotides were centrifuged for thirty seconds and dissolved in DEPC-H_2_O (RNase free water) before use. The template DNA **S**_**1**_ was denatured at 95 °C for 5 min in NEbuffers 2, followed by cooling to room temperature for annealing. Total volume reaction solution was 50 μl, containing template **S**_**1**_, 50 nM primer **S**_**2**_, 100 nM **S**_**5**_, 500 μM dNTP mixture, 10 U Nb.BbvCI, 20 U Rnase Inhibitor, 5 U Klenow Fragment (exo-) polymerase, 1 × NEBuffer 2 and different concentration target microRNA. The reaction was performed at 37 °C for 60 min. Then heated to 80 °C and maintain 20 min to make enzymes to inactivate. Then cooled to room temperature slowly, 100 pmol circular template, 10 U phi29 DNA polymerase, 10 × phi29 DNA polymerase buffer, and 5 μL dNTP mixture were added. The total reaction volume was 90 μL. The rolling circular amplification reaction was performed at 37 °C for 2 h. The reaction was terminated by incubation at 65 °C for 10 min. Then 10 μl 100 mM KCl and 1 μl 100 μM NMM were added and vibrated slowly at room temperature for 30 min.

### Measurement of Fluorescence Spectra

Measurement of Fluorescence Spectra was performed by Hitachi F-4600 fluorimeter (Tokyo, Japan). The fluorescence measurement of SDA reaction products was at the excitation wavelength of 492 nm and the fluorescence intensity at 518 nm was used for data analysis. The fluorescence measurement of SDA-RCA reaction products was at the excitation wavelength of 399 nm and the fluorescence intensity at 610 nm was used for data analysis.

### Gel Electrophoresis

The strand displacement amplification products were analyzed by 10% native polyacrylamide gel electrophoresis (PAGE) in 1 × TBE buffer at 100 V constant voltage at room temperature for 1 h. The amplification products of amplification. The total reaction products were analyzed by 1% agarose gel electrophoresis in 1 × TAE buffer at a 110 V constant voltage at room temperature for 1 h. The gels were stained by 4S Red Plus Nucleic Acid Stain. The imaging of gel was performed using WD-9413B Gel Imaging Systems.

## Additional Information

**How to cite this article**: Zheng, X. *et al*. Label-free detection of microRNA based on coupling multiple isothermal amplification techniques. *Sci. Rep.* **6**, 35982; doi: 10.1038/srep35982 (2016).

## Supplementary Material

Supplementary Information

## Figures and Tables

**Figure 1:**
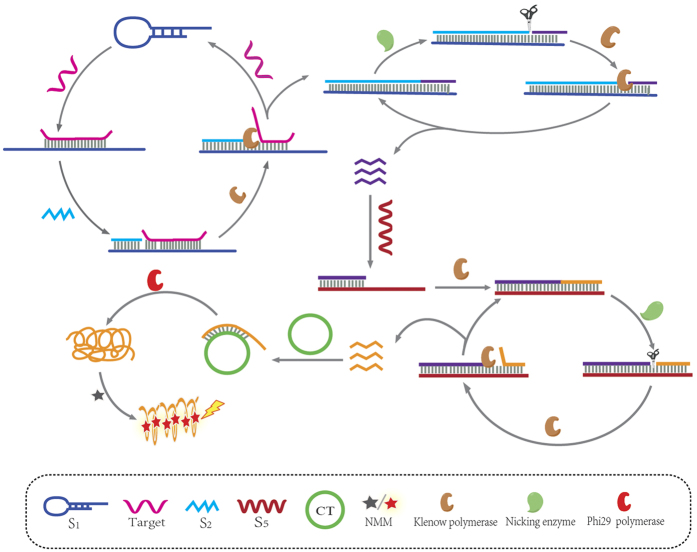
Schematic illustration of miRNA detection based on multiple amplification.

**Table 1:** Detection let-7d in human serum samples (The times of Parallel experiments is 3).

Sample	Added/pM	Measured/pM	Recovery/%	RSD%
1	1	0.93	93.0	1.56
2	10	10.52	105.2	2.05
3	100	96.81	96.81	1.97
4	500	510.3	102.1	2.62
