# Supplementary material for: Label-free detection of microRNA based on coupling multiple isothermal amplification techniques
Source: Sci Rep. 2016 Oct 25;6:35982. doi: 10.1038/srep35982 (PMC5078768; doi:10.1038/srep35982)
Supplement: Supplementary Information [file srep35982-s1.pdf]

Supporting Information

Label-free detection of microRNA based on coupling multiple isothermal amplification techniques

Xiangjiang Zheng[1,2], Li Niu[2], Di Wei[2], Xuemei Li[2] & Shusheng Zhang[1]

[1]Key Laboratory of Sensor Analysis of Tumor Marker, Ministry of Education, College of Chemistry and Molecular Engineering, Qingdao University of Science and Technology, Qingdao 266042, P. R. China.

[2]Shandong Provincial Key Laboratory of Detection Technology for Tumor Makers, Research Institute of Biochemical Analysis, Linyi University, Linyi 276000, P. R. China.

Xiangjiang Zheng (E-mail: zxj4408@126.com)

Li Niu (E-mail: lniu@ciac.jl.cn)

Di Wei (E-mail: dw344@cam.ac.uk)

Xuemei Li * (xuemei_li@yeah.net)

Shusheng Zhang* (E-mail: shushzhang@126.com)

*Corresponding author. Tel: + 86 539 8766867; fax: + 86 539 8766867.

E-mail address: xuemei_li@yeah.net (Xuemei Li)

shushzhang@126.com (Shusheng Zhang)

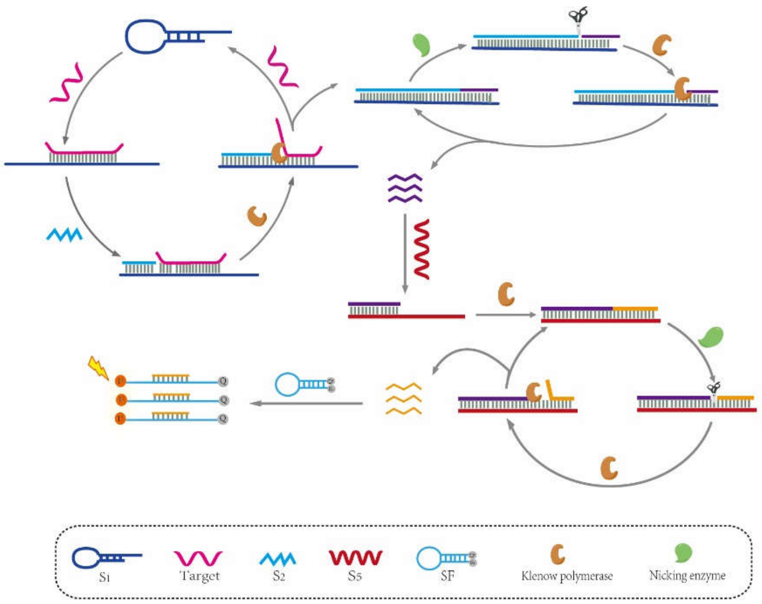

Figure S1 Schematic illustration of miRNA detection based on SDA reaction

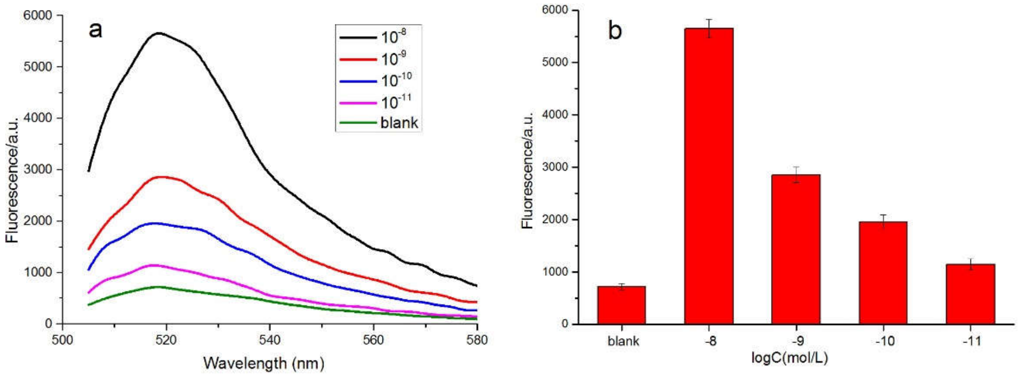

Figure S2 (a) Fluorescence spectra based on SDA reaction under different concentration of target miRNA. (b) Fluorescence intensity of different concentration of target miRNA with wavelength of 518nm.

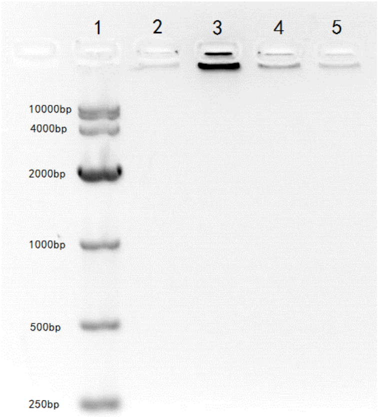

Figure S3 1% agarose gel electrophoresis of multiple amplification reaction. Lane 1: 10K marker, lane 2: absence of target, lane 3: the concentration of target is 10^{-11} M, lane 4: the concentration of target is 10^{-12} M, lane 5: the concentration of target is 10^{-13} M.

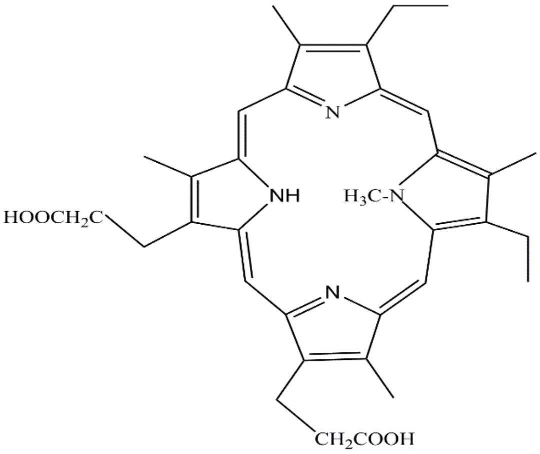

Figure S4 Molecular structure of NMM.

Table S1. The detection limit comparison between our method and other reported ones

Assay	Indicator or amplification	Detectionlimit	ref
Electrochemistry	using a hemin-G-quadruplex complex as the sensing elemen*t*	4 pM	S1
Colorimetry	Colorimetric sensing method onthe basis of the plasmoniccoupling effect	1 pM	S2
Electrochemistry	catalyzed hairpin assembly reaction (CHA) and hybridization chain reaction (HCR)	10 fM	S3
SERS	using a label-free multifunctional probe	6.3 fM	S4
Fluorescence	Using bifunctional strand displacement amplification mediated hyperbranched rolling circle amplification	0.18 pM	S5
Fluorescence	using isothermal gene amplification and graphene oxide	0.4 pM	S6
Colorimetry	coupling catalytic hairpin assembly	0.68 fM	S7
Fluorescence	Synergetic isothermal quadratic DNA machine	0.1 pM	S8
Fluorescence	This method	0.15 pM	

Reference

S1. Zhou, Y. L., Wang, M., Meng, X. M., Yin, H. S. & Ai, S. Y. Amplified electrochemical microRNA biosensor using a hemin-G-quadruplex complex as the sensing element. RSC Advances. **2**, 7140-7145 (2012).

S2. Park, J. Y. & Yeo, J. S. Colorimetric detection of microRNA miR-21 based on nanoplasmonic core–satellite assembly. Chem. Commun. **50**, 1366-1368 (2014).

S3. Cheng, Y., Lei, J. P., Chen, Y. L. & Ju, H. X. Highly selective detection of microRNA based on distance-dependent electrochemiluminescence resonance energy transfer between CdTe nanocrystals and Au nanoclusters. Biosens. Bioelectron. **51**, 431-436 (2014).

S4. Zhang, H., Liu, Y., Gao, J. & Zhen, J. H. Sensitive SERS detection of miRNA using a label-free multifunctional probe. Chem. Commun. **94**, 16836-16839 (2015).

S5. Zhang, L. R., Zhu, G. C. & Zhang, C. Y. Homogeneous and label-free detection of microRNAs using bifunctional strand displacement amplification-mediated hyperbranched rolling circle amplification. Anal. Chem. **86**, 6703−6709 (2014).

S6. Hong, C., Baek, A., Hah, S. S., Jung, W. & Kim, D. E. Fluorometric detection of microRNA using isothermal gene amplification and graphene oxide. Anal. Chem. **6,** 2999-3003 (2015).

S7. Zhang, J. Y., Lai, W. Q., Chen, G. N. & Tang, D. P. A rolling circle amplification-based DNA machine for miRNA screening coupling catalytic hairpin assembly with DNAzyme formation. Chem. Commun. **50**, 2935-2938 (2014).

S8. Zhang, Q., Chen, F., Xu, F., Zhao, Y. X. & Fan, C. H. Target-triggered three-way junction structure and polymerase/nicking enzyme synergetic isothermal quadratic DNA machine for highly specific, one-step, and rapid microRNA detection at attomolar level. Anal. Chem. **86**, 8098−8105 (2014).
